# Unsupervised Mining of HLA-I Peptidomes Reveals New Binding Motifs and Potential False Positives in the Community Database

**DOI:** 10.3389/fimmu.2022.847756

**Published:** 2022-03-21

**Authors:** Chatchapon Sricharoensuk, Tanupat Boonchalermvichien, Phijitra Muanwien, Poorichaya Somparn, Trairak Pisitkun, Sira Sriswasdi

**Affiliations:** ^1^ Center of Excellence in Computational Molecular Biology, Faculty of Medicine, Chulalongkorn University, Bangkok, Thailand; ^2^ Medical Sciences, Faculty of Medicine, Chulalongkorn University, Bangkok, Thailand; ^3^ Center of Excellence in Systems Biology, Faculty of Medicine, Chulalongkorn University, Bangkok, Thailand; ^4^ Research Affairs, Faculty of Medicine, Chulalongkorn University, Bangkok, Thailand

**Keywords:** HLA class I, *De novo* peptide sequencing, HLA binding motifs, HLA peptidomics, IEDB

## Abstract

Modern vaccine designs and studies of human leukocyte antigen (HLA)-mediated immune responses rely heavily on the knowledge of HLA allele-specific binding motifs and computational prediction of HLA-peptide binding affinity. Breakthroughs in HLA peptidomics have considerably expanded the databases of natural HLA ligands and enabled detailed characterizations of HLA-peptide binding specificity. However, cautions must be made when analyzing HLA peptidomics data because identified peptides may be contaminants in mass spectrometry or may weakly bind to the HLA molecules. Here, a hybrid *de novo* peptide sequencing approach was applied to large-scale mono-allelic HLA peptidomics datasets to uncover new ligands and refine current knowledge of HLA binding motifs. Up to 12-40% of the peptidomics data were low-binding affinity peptides with an arginine or a lysine at the C-terminus and likely to be tryptic peptide contaminants. Thousands of these peptides have been reported in a community database as legitimate ligands and might be erroneously used for training prediction models. Furthermore, unsupervised clustering of identified ligands revealed additional binding motifs for several HLA class I alleles and effectively isolated outliers that were experimentally confirmed to be false positives. Overall, our findings expanded the knowledge of HLA binding specificity and advocated for more rigorous interpretation of HLA peptidomics data that will ensure the high validity of community HLA ligandome databases.

## Introduction

Human leukocyte antigen (HLA) is a family of proteins in the immune system that binds to and presents peptide fragments of proteins expressed in the body for recognition by T cells. Peptides that form stable complexes with HLA proteins are also called HLA ligands. When a foreign antigen, whose amino acid sequence differs from the host’s proteome, was intracellularly processed and presented on the cell surface by HLA proteins, the cell containing foreign antigen would be recognized T cell and subsequently destroyed by the immune system. Therefore, HLA-peptide binding activity has been extensively studied for medical and biotechnology applications in vaccine design and cancer immunotherapy (1–6).

HLA class I is a subclass of the HLA system that recognizes peptides with 8-15 amino acids in length. The binding affinity of a peptide to an HLA class I molecule mainly depends on an 8- to 10-residue motif on the peptide including a few HLA allele-specific amino acid residues at anchor positions (7–10). Other residues on the peptide are relatively unconstrained, but some amino acid combinations can affect the binding affinity. To date, although a few works have highlighted the multiple specificities of HLA class I binding (8, 11, 12) and HLA class II binding (13), the motif of each HLA class I allele is still represented with a single amino acid frequency profile in major databases (14, 15). In other words, HLA class I motifs were assumed to be unimodal. While this simplification may not have a noticeable impact on the development of HLA binding prediction models (11, 16), it may limit the design landscape of vaccines if researchers use only the consensus motif as a guideline.

Breakthroughs in HLA peptidomics, which enabled the isolation of HLA proteins from the cell surface followed by high-throughput sequencing of HLA ligands, have cataloged a large amount of ligand sequences for a multitude of HLA class I and class II alleles from both cell lines and patient samples (8, 10, 17, 18). These data accelerated the improvement in HLA binding prediction accuracy as well as enabled detailed characterization of HLA binding specificity. HLA peptidomics is also being increasingly utilized to identify tumor-specific or tumor-elevated antigens in cancer patients, which can then be developed into a cancer vaccine to boost the immune system to target cancer cells (5, 6). Nonetheless, results from HLA peptidomics only indicate whether the peptides are bound to the HLA proteins and presented on the cell surface but provides no information on their actual binding affinities. Hence, downstream analyses of HLA peptidomics often involve HLA binding affinity predictions by artificial neural network models to screen for peptides with strong bindings. Furthermore, like most mass spectrometry analyses, results from HLA peptidomics can include contaminants such as carry-over peptides and non-HLA-specific proteolytic peptides or artifacts from in-source fragmentations (19, 20). Immunoaffinity purification of HLA proteins can also introduce non-specific co-isolates (21). A few studies have proposed additional analysis steps that would help reduce the number of contaminant identifications originating from these sources (20, 21).

Increasing the understanding of HLA binding specificity and the quality of known HLA ligand databases is crucial for designing better vaccines against constantly emerging pathogens and improving the accuracy of HLA binding and immunogenicity predictions. In this study, a hybrid *de novo* peptide sequencing strategy with SMSNet (22) was applied to large-scale HLA class I peptidomics datasets (8, 17) to uncover new candidate HLA ligands that would expand the existing databases. Subsequent unsupervised clustering of known and newly discovered ligands for each HLA class I allele strongly suggested that several alleles recognize multiple, clearly distinct motifs. Many potential false positives whose sequences do not match the corresponding HLA binding motifs were also observed. A validation experiment confirmed that almost all potential false positives exhibit no HLA binding activity. Most importantly, many of these false positives were also found in the Immune Epitope Database (15) and could be erroneously used by the community. Additionally, our HLA peptidomics analysis of a B-lymphoblastoid cell line expressing both HLA class I and class II alleles highlighted the capability of SMSNet to identify high-affinity antigens in a multi-allelic setting.

Overall, our work revisited two key aspects of the HLA study: the representation of the HLA binding motif and the interpretation of HLA peptidomics data. The findings strongly suggested that the implicit unimodal assumption of HLA class I motifs should be replaced by a multimodal representation and that the quality of HLA peptidome-derived HLA-I ligands reported in the community database may be questioned.

## Results

### Re-Analysis of Large-Scale Mono-Allelic HLA Class I Peptidomes

**Figure 1:**
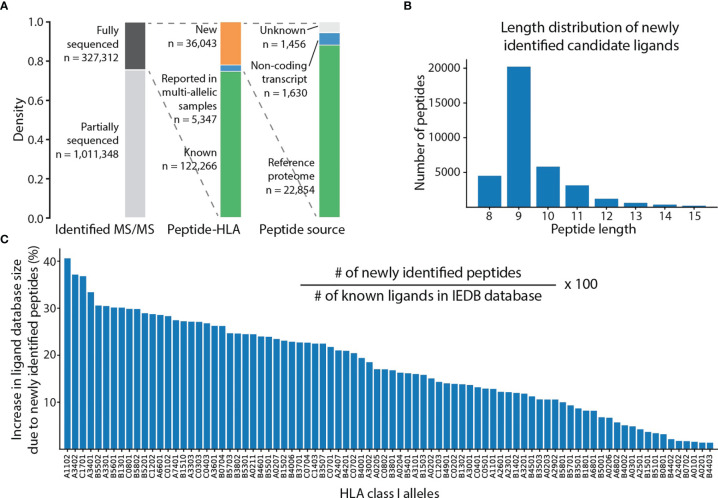
SMSNet identified a large number of new ligands from public HLA peptidomics datasets. **(A)** Statistics of MS/MS spectra, peptide-HLA pairs, and the sources of peptides identified by SMSNet on mono-allelic HLA peptidomics datasets of 88 HLA class I alleles (see Methods). **(B)** Length distribution of all identified peptides. **(C)** Potential increase in the size of the database of known ligands from this study, assuming that all newly identified sequences are true ligands. The number of known ligands for each allele was extracted from the IEDB database by counting unmodified antigens and antigens with major modifications, namely oxidized methionine and phosphorylated serine, threonine, and tyrosine.

*De novo* peptide sequencing with SMSNet (22) was shown to be effective for discovering new candidate HLA class I antigens from a peptidomics dataset. Here, SMSNet was applied to a larger collection of high-quality HLA peptidomics data from mono-allelic human B lymphoblastoid cell lines encompassing 88 HLA-A, -B, -C, and -G alleles (8, 17). In total, 109,372 unique peptide sequences with lengths ranging from 8 to 15 amino acids were identified from 327,312 mass spectra (**Figure 1A**, **Supplementary Table 1**). There are 36,043 newly discovered peptide-HLA pairs involving 25,718 unique peptide sequences as well as 5,347 additional pairs that have been previously observed in multi-allelic patient samples. Over 88% (22,854 peptides) of newly discovered peptides could be mapped to the human reference proteome. About half of peptides with unknown origins could be traced to open reading frames on non-coding transcripts (1,630 peptides). The length distribution of 25,718 newly identified peptides matches well with past observations (23), with the majority being 9-mers (**Figure 1B**). Most importantly, the discovery of 36,043 new peptide-HLA pairs has the potential to expand the database of known HLA class I ligands by up to 35-40% for some major alleles such as HLA-A*11:02 and HLA-A*34:02 (**Figure 1C**).

### Extent of Tryptic Peptide Contaminations in HLA Peptidomics Data

**Figure 2:**
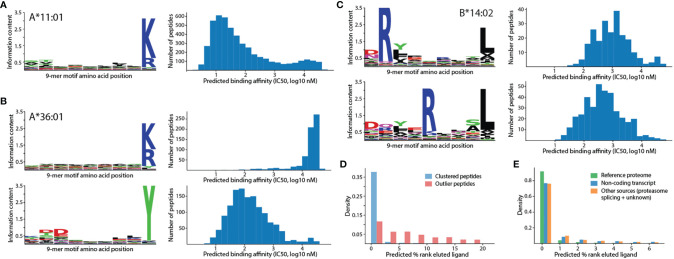
Unsupervised clustering revealed potential false positives and multiple motif specificities. **(A)** Single 9-mer motif identified for HLA-A*11:01 together with predicted binding affinities (IC50, nM unit). **(B)** Two motifs identified for HLA-A*36:01, one of which consists mainly of tryptic peptides and exhibits lower affinities (higher IC50 value indicates lower affinity). The top motif is expected to be a false positive. **(C)** Two distinct motifs identified for HLA-B*14:02 with arginine at different residue positions but similar predicted affinities. **(D)** Distributions of predicted percentage rank (% rank) of eluted ligand for clustered peptides and outlier peptides. A higher % rank indicates lower binding affinity. Bin size is 2%. **(E)** Distributions of predicted percentage rank of eluted ligand for peptides from various sources. Bin size is 1%.

Past analyses of HLA peptidomics were careful not to report 9-mer tryptic peptides as antigens for HLA alleles whose binding motifs do not end with an arginine or a lysine (10). Among 88 HLA class I alleles investigated in this study, 12 have binding motifs ending with an arginine or a lysine (**Figure 2A**, HLA-A*03:01, HLA-A*11:01, HLA-A*11:02, HLA-A*30:01, HLA-A*31:01, HLA-A*33:01, HLA-A*33:03, HLA-A*34:01, HLA-A*34:02, HLA-A*66:01, HLA-A*68:01, and HLA-A*74:01), and are expected to present tryptic peptides. However, for the other 76 alleles, we also identified 2,838 tryptic peptides from the monoallelic peptidomics data (**Supplementary Table 1**) that were assigned as true ligands to the same alleles in the Immune Epitope Database (IEDB) (15). Motif clustering with GibbsCluster (24) and binding affinity prediction with NetMHCpan (25) clearly illustrated that these tryptic peptides form separate clusters with much lower binding affinities than the known motifs (**Figure 2B** and **Supplementary Figure 1**). Overall, clusters of tryptic peptides identified from the monoallelic peptidomics data were observed for 11 out of 76 alleles whose binding motifs do not end with an arginine or a lysine. In all 11 alleles, more than 13% of identified peptides are tryptic. Considerable but lower extent of tryptic peptide contamination were also previously reported for these alleles (8), in which 9-28% of identified peptides are tryptic (**Supplementary Table 2**).

To test whether tryptic peptides identified alleles whose motifs do not end with an arginine or a lysine are specifically recognized by the corresponding alleles, and thus may be true ligands, predicted binding affinities for the observed tryptic peptide-HLA allele pairs were compared with the predicted binding affinities between random pairs. This revealed that almost all alleles do not exhibit stronger affinities toward the identified tryptic peptides than toward random tryptic peptides (**Supplementary Figure 2**). Hence, these tryptic peptides are likely to be contaminants. It should be noted that predicted binding affinities from NetMHCpan can separate HLA alleles that are expected to present tryptic peptides from other alleles (**Supplementary Figure 3**). Furthermore, even among 12 HLA alleles that are expected to present tryptic peptide, the presence of two modes in the distributions of predicted binding affinities for their tryptic ligands (**Supplementary Figure 3**, HLA-A*11:01 and HLA-A*34:02 in particular) suggests that some identified tryptic peptides for these alleles may yet be false positives. A detailed motif analysis of tryptic peptides belonging to these two modes showed that tryptic peptides with strong binding affinities (predicted IC50 ≤500 nM) exhibit additional enrichments of specific amino acids at the first and second motif positions while tryptic peptides with weak binding affinities (predicted IC50 >500 nM) do not exhibit any pattern.

### HLA Alleles With Multiple Binding Motifs

In addition to revealing clusters of false-positive tryptic peptides, unsupervised motif clustering of peptides identified from monoallelic peptidomics data also showed that several HLA class I alleles possess multiple motif specificities that cannot be explained by length alone (11). For example, HLA-B*14:02 peptides contain arginine exclusively at either the 2^nd^ or the 5^th^ position of the motif with only slight differences in predicted binding affinities (**Figure 2C**, average predicted affinities are 2,067 nM and 1,733 nM, respectively). These low predicted binding affinities, despite being associated with clear motif patterns, may reflect the limitation of binding affinity prediction as only 65 out of more than 1,800 HLA-B*14:02 ligands on IEDB contain quantitative binding affinity data that can be used to train the prediction model. The motif for this allele was previously reported as a combined pattern with arginine at both positions (10, 14). Other alleles with multiple, clearly distinct motifs include HLA-B*15:01, HLA-B*51:01, and HLA-B*53:01 (**Supplementary Figure 4**). Additionally, several alleles also contain multiple related motifs that differ only by the shift of the anchor residue at the 2^nd^ position to the 1^st^ position (**Supplementary Figure 5**), which can be explained as mixtures of 9-mer and 10-mer motifs (**Supplementary Figure 6**). The fact that most motifs consist of peptides with similar, intermediate predicted binding affinities further illustrated the limitation of binding affinity prediction and the power of unsupervised analyses for discovering new biological insights into HLA binding specificities.

### False Positives in HLA Peptidomics Data

A by-product of unsupervised motif clustering is the designation of outlier peptides that do not fit into any motif. Here, a peptide is labeled as an outlier if the quality of the motif clustering, as measured by Kullback-Liebler distance in GibbsCluster, is improved by removing the peptide from the analysis. This result revealed that up to 5-6% of peptides identified from monoallelic peptidomics data were classified as outliers for some HLA alleles (e.g., HLA-B*14:02 and HLA-A*02:05, **Supplementary Table 2**). As expected, the predicted binding percentage ranks of these outliers were much higher than those of peptides belonging to motif clusters (**Figure 2D**, higher percentage rank indicates weaker binding affinity). More than 83.8% and 95.5% of outliers do not pass the 2% rank threshold for weak binder and the 0.5% rank threshold for strong binder (25), respectively. In contrast, only 10.2% and 20.4% of peptides that belong to motif clusters failed the same thresholds. Among peptides of unknown origins that were identified solely by *de novo* sequencing, more than 47% of them pass the 0.5% rank threshold for strong binder (**Figure 2E**).

**Figure 3:**
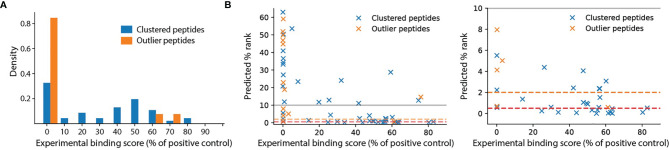
HLA binding assay for HLA-B*14:02. Peptide synthesis and binding assay were performed by ProImmune, Ltd. (see Methods). **(A)** Distributions of binding scores, measured as the percentages of the binding activity compared to a positive control, for clustered peptides (n = 46) and outlier peptides (n = 13). **(B)** Comparison of predicted percentage ranks of eluted ligand (% rank) and binding scores. The orange and red dashed lines indicate the 2% rank and 0.5% rank thresholds for weak and strong binders, respectively. The left panel shows the full range of % rank while the right panel shows the zoomed-in at % rank below 10%.

To test whether outlier peptides identified by unsupervised motif clustering are false positives or true ligands with very weak binding affinity, we performed an HLA binding assay on 59 newly identified antigens for HLA-B*14:02 (**Supplementary Table 3**, 13 outliers and 46 non-outlier peptides). This assay showed that all outlier peptides except LRNGGHFVI and LPFCRPGPEGQL exhibited almost no binding activity against the HLA molecules (**Figure 3A**, relative binding activity <1% of positive control). The high binding affinity of LRNGGHFVI and LPFCRPGPEGQL may be attributed to the arginine residues. LRNGGHFVI was likely called an outlier because its non-arginine residues did not fit the motif profile of HLA-B*14:02 (**Figure 2C**, top cluster). For LPFCRPGPEGQL, this peptide was likely called an outlier because the middle arginine residue was not predicted to take part in the 9-mer binding motif by NetMHCpan (the predicted core motif was LPFGPEGQL). Overall, the experimental binding result is in good agreement with computational affinity prediction (**Figure 3**, Spearman’s rank correlation = –0.62 with p-value = 1.6e-7). These pieces of evidence together strongly suggest that the majority of outlier peptides are false positives.

### Application of SMSNet on Multi-Allelic Peptidomics Data

To showcase the capability of SMSNet in a multi-allelic setting, we performed an HLA class I and an HLA class II peptidomics experiments on a B-lymphoblastoid cell line (BLCL1408-1038) expressing HLA-A*01:01, HLA-B*08:01, HLA-C*07:01, HLA-DPA1*01:03, HLA-DPB1*04:01/02:01, HLA-DQA1*05:01/05:01, HLA-DQB1*02:01/02:01, and HLA-DRB1*03:01/03:01. Both SMSNet and PEAKS-DB (26, 27), which rely on the same principle of first performing *de novo* peptide sequencing and then allowing low-confidence amino acids to be refined *via* a subsequent database search, were used to analyze the data. As each tool was optimized differently, the confidence thresholds for peptide identification were set separately (see Methods). Each peptidomics sample was processed twice through the mass spectrometer with slightly different settings on the accepted precursor charge states: one accepting all precursors and another accepting only precursors with 2+ or higher charge state. NNAlign_MA (28) was used to predict the binding probabilities for each identified antigen simultaneously against all HLA class I or class II alleles present and assign each peptide to the most likely allele. The maximum predicted binding score was taken for each peptide. Overall, the lengths of identified peptides closely followed the expected ranges, with the highest frequency of 9-mer for HLA class I peptides and 15-mer for HLA class II peptides (**Supplementary Figure 7**). Peptides assigned to HLA-A*01:01 or HLA-B*08:01 matched known motifs for the respective alleles, while only 36 unique peptides were identified for HLA-C*07:01 (**Supplementary Figure 7**). Across technical replicates, SMSNet identified the same set of peptides slightly more consistently than PEAKS-DB (**Supplementary Table 4**, 45.9% versus 34.5% for HLA class I peptides, and 29.3% versus 15.1% for HLA class II peptides).

**Figure 4:**
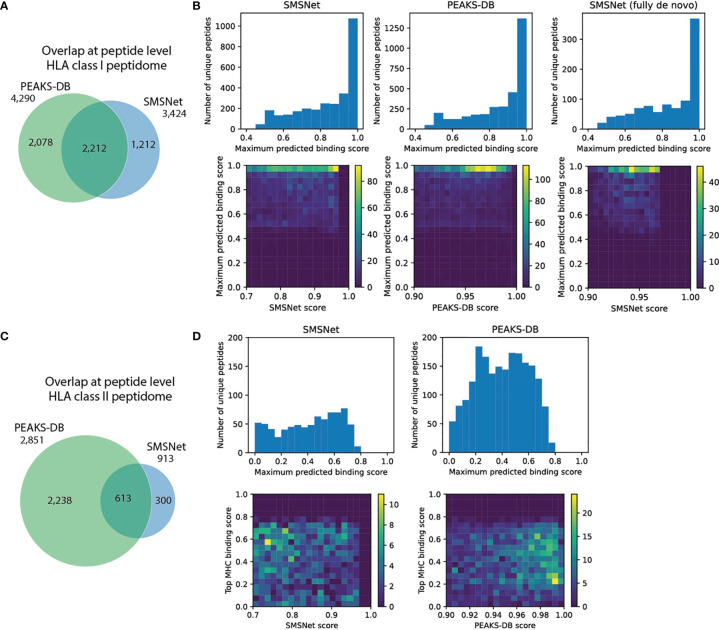
Comparison of SMSNet and PEAKS-DB on multi-allelic B-lymphoblastoid sample. **(A)** Overlap of identified peptides from HLA class I peptidomes between SMSNet and PEAKS-DB. **(B)** Histograms show the distributions of predicted binding scores, calculated as the maximum score over HLA-A*01:01, HLA-B*08:01, and HLA-C*07:01, which are expressed in the cells, for peptides identified by each tool. Heatmaps show the association between predicted binding scores and peptide identification confidence scores reported by each software. Histogram and heatmap for peptides identified fully *de novo* by SMSNet (i.e., without relying on database search step) are also shown separately. **(C)** Overlap of identified peptides from HLA class II peptidomes between SMSNet and PEAKS-DB. **(D)** Similar histograms and heatmaps as in **(B)** for HLA-DPA1*01:03, HLA-DPB1*04:01/02:01, HLADQA1*05:01/05:01, HLA-DQB1*02:01/02:01, and HLADRB1*03:01/03:01, which are expressed in the cells.

For HLA class I peptidome, SMSNet and PEAKS-DB had a 40% overlap at peptide level (**Figure 4A** and **Supplementary Table 5**) and agreed on the same peptides for 98% of the MS/MS spectra identified by both tools (2,170 of 2,215 spectra). To assess the quality of peptides identified by each tool, predicted HLA binding scores and peptide identification confidence scores were visualized together. Tools that identified peptides with high HLA binding scores with high confidences should be preferable. This analysis revealed that both SMSNet and PEAKS-DB identified peptides with high predicted binding probabilities and high confidences (heatmaps in **Figure 4B**). Furthermore, peptides identified fully *de novo* by SMSNet also exhibit the same level of quality as peptides that passed a follow-up database search step (**Figure 4B**, rightmost panel). It should be noted that all methods identified peptides whose lengths do not match the expected lengths of HLA class I ligands (8-15 amino acids), which were removed from consideration, and that peptides with post-translational modifications were not considered here as their binding probabilities could not be predicted.

For the HLA class II peptidome, both SMSNet and PEAKS-DB made fewer identifications (913 and 2,851 versus 3,424 and 4,290 peptides) and had smaller overlap (19% versus 40% at peptide level) than HLA class I peptidome’s counterpart (**Figure 4C**). This finding is likely because the yields of HLA class II immunoaffinity purification were lower and partly because HLA class II antigens are longer and consequently slightly harder to confidently identify from MS/MS spectra. Nonetheless, the two tools still agreed on the same peptides for 94% of the MS/MS spectra identified by both (355 of 377 spectra) and 9 of 22 disagreements occurred due to difference in interpretation between deamidation of glutamine and asparagine versus glutamic acid and aspartic acid. Here, PEAKS-DB was able identify significantly more peptides than SMSNet, likely because PEAKS-DB can identify long peptide more confidently. Peptides that were identified solely by PEAKS-DB were significantly longer than those identified in common with SMSNet (Mann-Whitney U test p-value <1e-10), with the longest identified peptide being 48 amino acids in length. On the other hand, the longest peptide identified by SMSNet is only 27 amino acids in length. In terms of the predicted binding scores, peptides identified by SMSNet exhibited slightly higher scores than PEAKS-DB’s (**Figure 4D**, Mann-Whitney U test p-value = 0.0131). But as most of the predicted binding probabilities were quite low, it is inconclusive whether one tool is better than the others based on this aspect.

### Applying Unsupervised Analyses on Multi-Allelic Peptidomics Data

To illustrate how our proposed unsupervised analysis can be applied to a newly generated peptidomics dataset, motif clustering, binding affinity prediction, and tryptic peptide identification were applied to the newly generated B-lymphoblastoid (BLCL1408-1038) HLA class I peptidomes. Peptides identified by SMSNet and PEAKS-DB were aggregated and analyzed together. Overall, NNAlign_MA assigned 1,795 peptides to HLA-A*01:01, 2,598 peptides to HLA-B*08:01, and 36 peptides to HLA-C*07:01 (**Supplementary Figure 7**). To handle the situation where only a few peptides were identified for an allele, as is the case for HLA-C*07:01 here, all reported ligands from the IEDB database were also included during the unsupervised analyses to help establish motif clusters. Motif clustering with GibbsCluster flagged 112 peptides as potential outliers, 108 of which were assigned to HLA-A*01:01. Visualization of the motif profiles and predicted binding affinities strongly suggests that these outliers are false positives, with complete absence of anchor amino acids and very low affinities (**Supplementary Figure 8**). It should be noted that flagged outliers and tryptic peptides could account for most of the peptides with low predicted binding affinities (IC50 > 30,000 nM). For HLA-B*08:01, although only 3 peptides were flagged as outliers, one of the motif clusters suggested by GibbsCluster for this allele could consist of false positives because its member peptides exhibited much lower predicted binding affinities compared to other clusters’ (IC50 >3,000 nM versus <1,000 nM) together with a lack of the anchor lysine or arginine residue at the 5^th^ position (**Supplementary Figure 9**, top panel). In terms of tryptic peptide contaminant, only 9 peptides are fully tryptic, and 56 peptides are partial tryptic.

## Discussion

Our work highlighted the need for a careful downstream analysis of peptides identified from the HLA peptidomics experiment to remove potential false positives. Although a prior work has provided detailed analyses to account for non-ligand contaminants (20), there are still true peptide identifications that bind very weakly or non-specifically to the target HLA allele. Inclusion of these peptides as true HLA ligands in community database can potentially mislead researchers as HLA peptidome-derived peptides are not accompanied with binding affinity values. Unsupervised clustering of identified putative HLA ligands not only elucidate allele-specific binding motif patterns (11, 12) but also revealed clusters of tryptic peptides for HLA alleles that should not recognize an arginine or a lysine at the C-terminus of the binding motif (**Supplementary Figure 1**) as well as outlier peptides that do not fit into any cluster. A small-scale HLA binding experiment of putative ligands of HLA*B14:02 confirmed that almost all outliers (11 of 13) exhibited no binding activity (**Figure 3A**, relative affinity < 1% of positive control) while 72% (33 of 46) of non-outliers exhibited some binding activities. Outlier peptides are also predicted to be weaker binders than *de novo*-identified peptides whose origins cannot be verified (**Figures 2D, E**, NetMHCpan % rank eluted ligand). Similarly, most tryptic peptides are likely false positives because their predicted binding affinities are not stronger than those between random tryptic peptides and HLA alleles (**Supplementary Figure 2**). It is interesting to note that both motif patterns and predicted binding affinities have to be analyzed together to clearly distinguish between multiple motif specificities and outliers. When multiple motifs were identified with similar binding affinity distributions (**Supplementary Figures 4 and 5**), they can indicate multiple motif specificities for the allele. Yet, when the distributions of predicted binding affinities were also bimodal (**Supplementary Figure 1**), they indicate potential outliers.

Overall, there are 3,846 potential false positives identified here that have been reported as positive antigens in the IEDB database. Although this number may seem small compared to the current size of the IEDB database (>300,000 allele-specific antigens), the presence of potential false positives is considerable for HLA alleles with fewer known ligands. For example, 23% (679 of 2,957), 16% (342 of 2,165), and 11% (209 of 1,843) of IEDB reported ligands for HLA-C*03:03, HLA-A*36:01, and HLA-B*57:01, respectively, are flagged as potential false positives here. Furthermore, the bimodal distribution of predicted affinities suggested that there are more false positives among peptides that belong to motif clusters (**Figure 2A**). Hence, careful analysis of both future HLA peptidomics data and the data already deposited into the IEDB database is needed in order to maintain the integrity of community antigen databases and prevent errors from propagating into HLA binding prediction and immunogenicity prediction models. A first step for cleaning IEDB entries that were derived from peptidomics data would be to flag all tryptic peptides for HLA alleles whose binding motifs do not end with a lysine or an arginine. Each sequence that can be mapped to a known protein should also be checked for a flanking arginine or lysine in the protein sequence to determine whether it is fully tryptic, and more likely to be a contaminant. If these tryptic peptides originated from common contaminants or highly expressed proteins, they could be potentially removed. Otherwise, their predicted binding affinities should be examined. Next, unsupervised analyses of motifs clusters and predicted binding affinities performed here should be repeated on the entirety of IEDB database to flag potential outliers. Adding potential tryptic contaminant and outlier labels to IEDB entries could caution future users and developers of prediction models.

Our unsupervised framework, which focuses on motif specificity and binding affinity distributions, perfectly complements the existing best practices (20) for screening true HLA ligands from peptidomics data that focus more on the proteolytic cleavages, chromatographic carryover, in-source fragmentation, and other factors that affect the quality of identification. Intriguingly, when we applied some of the best practices on our analysis of mono-allelic HLA peptidome data, the protein and peptide coverage ratios, which would support the possibility of proteolytic cleavage, were much lower than those previously reported (20) (**Supplementary Figure 10**, top panels). For example, the coverage of beta actin (ACTB) protein, which was found to be as high as 39.27% in another dataset (20), is only 2.83% here. Other proteins with high coverages (up to 4.32%) include three 40S ribosomal proteins (RPS3, RPS19, and RPS23) and two histones (H4C1 and H3-3B) which are known to be highly expressed as expected. This indicates that the thresholds for identifying proteolytic peptides need to be re-tuned for each study. On the other hand, while no potential proteolytic peptide was identified, 796 peptide-HLA allele pairs could be flagged as putative products of in-source fragmentation (**Supplementary Figure 10**, bottom panels). The low average (geometric mean) predicted binding affinities for these peptides at 7,706 nM suggested that they might be false positives. Only 16 of these were already designated as outliers by our analysis and 510 were reported as true ligands in IEDB. These findings highlight the orthogonality between this work and existing best practices and strongly suggest that they should be performed in conjunction on future HLA peptidomics studies.

It is interesting to note that this work and prior unsupervised clustering analyses of the same HLA class I alleles (11, 12) do not always identify the same multiple motif specificities. For example, three motifs were identified for HLA-B*15:01 here (**Supplementary Figure 4**) but not in prior analysis (11). On the other hand, three motifs for HLA-B*07:02 were previously reported (12), but only a single motif was identified here. This latter case is especially unexpected because the motif identified here was not the one with the highest number of associated peptides among the three reported motifs. As a quality control, both motifs of HLA-B*51:01 (**Supplementary Figure 4**) were consistently identified (11). In addition to multiple specificities, related motifs that differ by a shift of the 2^nd^ residue position to the 1^st^ residue position, with only minor changes in predicted binding affinities, were observed in several alleles (**Supplementary Figure 5**). These indicate the presence of 10-mer or longer motif patterns that were truncated to 9-mer during the core binding motif prediction by NetMHCpan (**Supplementary Figure 6**). Additionally, unsupervised clustering was also able to capture minor inter-residue cooperation between non-anchor positions and represent them in separate motif clusters (HLA-B*53:01 in **Supplementary Figure 4**, HLA-B*15:03 and HLA-B*40:01 in **Supplementary Figure 4**). However, it should still be noted that some peptides, including LRNGGHFVI and LPFCRPGPEGQL, that were flagged as outliers by our procedure turned out to be viable ligands. These incidences were partly due to our reliance on the core 9-mer motifs predicted by NetMHCpan, which missed the arginine residue in LPFCRPGPEGQL, but they also reflected that solely relying on computational analyses can lose promising ligands.

Our work also illustrated the capability of hybrid *de novo* sequencing with SMSNet to uncover new HLA antigens in both mono-allelic and multi-allelic peptidomics samples. More than 36,000 new peptide-HLA pairs were identified from public mono-allelic HLA class I peptidomics datasets (8, 17) that have already been extensively analyzed. The new putative antigens could potentially expand the antigen pools for some HLA alleles by up to 40% (**Figures 1A, C**). SMSNet also exhibited good agreement with the *de novo*-assisted database search results from PEAKS-DB (94-98% of MS/MS spectra identified by both tools) on newly generated HLA class I and class II peptidomes of B-lymphoblastoid cell line (BLCL1408-1038), both producing peptide identifications with high predicted binding affinities to HLA class I alleles (**Figure 4B**). Furthermore, even in the absence of a reference proteome database, SMSNet was able to produce peptides with high predicted binding affinities. The fewer numbers of putative HLA class II antigens identified by SMSNet and PEAKS could be attributed to the lower yield of HLA class II immunoaffinity purification and partially to the increased peptide length. PEAKS-DB was able to produce many more identifications than SMSNet (**Figure 4C**) likely because it can identify longer peptides more reliably. Although predicted binding probabilities to HLA class II alleles were slightly higher for peptides identified by SMSNet, the low confidence of the predictions makes this result inconclusive and illustrates a current limitation in computational analysis of HLA class II binding. Nonetheless, combining results from multiple software tools is a well-established approach that has been shown to improve the quality of proteomics analyses (29, 30). From our results, it is clear that SMSNet and PEAKS could be used together to maximize the sensitivity of putative HLA antigen detection.

## Methods

### Cell Line and Antibody Preparation

B-lymphoblastoid cell line (BLCL1408-1038) expressing HLA-A*01:01, HLA-B*08:01, HLA-C*07:01, HLA-DPA1*01:03, HLA-DPB1*04:01/02:01, HLA-DQA1*05:01/05:01, HLA-DQB1*02:01/02:01, and HLA-DRB1*03:01/03:01 was purchased from Fred Hutchinson Cancer Research Center, Washington, USA. Cells were cultured in RPMI 1640 media supplemented with 10% fetal bovine serum, 50 U/ml penicillin in a humidified incubator at 37C with 5% CO_2_. Purified pan HLA-A, -B, -C and pan HLA-DR, -DP, -DQ antibodies were generated from W6/32 (ATCC, USA) and IVA12 (provided by the lab of Professor Anthony Purcell, Monash University, Australia) hybridoma cells cultured in RPMI 1640 media supplemented with 10% fetal bovine serum, 50 U/ml penicillin and expanded in roller bottles at 37C with 5% CO_2_. Secreted monoclonal antibodies were harvested from spent media and purified using Protein A resin with ÄKTA purification system (Cytiva, USA).

### Immunoprecipitation of HLA Class I and Class II Complexes

BLCL1408-1038 cell pellets (1 x 10^8^) were pulverised using an MM400 Retsch Mixer Mill (Retsch, Germany) and lysed with 0.1% IGEPAL CA-630, 100 mM Tris, 300 mM NaCl, pH 8.0 Complete Protease Inhibitor Cocktail (Roche, Switzerland). The supernatant was passed through a Protein G resin pre-column (500 μL) to remove non-specific binding materials. HLA class I and II immunoaffinity purification was performed as previously described (31). Briefly, the pre-cleared supernatant was incubated with 10 mg of pan HLA-A, -B, and -C antibodies or 10 mg of pan HLA-DR, -DP, and -DQ antibodies coupled to Protein G resin with rotation overnight at 4C. After conjugation, the resins were washed with 10 ml of ice-cold wash buffer 1 (0.005% IGEPAL, 50 mM Tris, pH 8.0, 150 mM NaCl, 5 mM EDTA), 10 ml of ice-cold wash buffer 2 (50 mM Tris, pH 8.0, 150 mM NaCl), and 10 ml of ice-cold wash buffer 3 (50 mM Tris, pH 8.0, 450 mM NaCl). Bound complexes were eluted from the column using 5 column volumes of 10% acetic acid. Eluted peptides were fractionated by reverse-phase high-performance liquid chromatography (Shimadzu, Japan) on a 4.6 mm diameter Chromolith SpeedROD RP-18 (Merck, USA). The optimized conditions were as follows: mobile phase A (0.05% v/v TFA, 2.5% v/v ACN in water), mobile phase B (0.045% v/v TFA, 90% v/v ACN in water), flow rate of 1 mL/minute, temperature of 30C, and injection volume of 200 μL. The elution program was set as follows: 0-5% of mobile phase B over 1 minute, 5-15% of mobile phase B over 4 minutes, 15-45% of mobile phase B over 30 minutes, 45-100% of mobile phase B over 15 minutes, and 100% of mobile phase B over 4 minutes. Fractions were collected in 1 mL each. Consecutive fractions were pooled into 11 fractions. Pooled fractions were concentrated by vacuum centrifugation and reconstituted in 0.1% FA.

### LC-MS/MS Analysis of HLA Peptidome

Pooled peptide fractions eluted from an HLA class I sample and an HLA class II sample were analyzed on a Q Exactive mass spectrometer (Thermo Fisher Scientific, USA) coupled to an EASY-nLC 1000 (Thermo Fisher Scientific, USA). Peptide samples were separated at a flow rate of 300 μL/minute of buffer B (80% ACN, 0.1% FA). The gradient was set at 4-20% of buffer B over 30 minutes, 20-28% of buffer B over 40 minutes, 28-40% of buffer B over 5 minutes, 40-95% of buffer B over 3 minutes, washing with 95% of buffer B over 8 minutes, re-equilibration with buffer A (2% ACN/0.1% FA) over 5 minutes. Mass spectra resolutions were set at 70,000 for full MS scans and 17,500 for MS/MS scans. The normalized collision energy for HCD fragmentation was set at 30%. The m/z scan range was set at 350-1,400. Dynamic exclusion was set at 15 seconds. For HLA class I samples, the maximum injection times were set at 120 ms for full MS scan and 120 ms for MS/MS scans. For HLA class II samples, the maximum injection times were set at 200 ms for full MS scan and 120 ms for MS/MS scans. Each sample was analyzed twice on the mass spectrometer, once where precursor charge states of +2 or higher were accepted (raw file names beginning with 2zup) and another where all charge states were accepted (raw file names beginning with 1zup).

### Collection of Published HLA Class I Peptidomics and Antigen Data

A combined dataset of mass spectrometry raw data of mono-allelic HLA class I peptidomes (399 raw files, 88 HLA alleles) were obtained from two prior studies (8, 17) (MSV000080527 and MSV000084172). List of reported antigen-HLA pairs were obtained from the Immune Epitope Database (15) (IEDB, downloaded December 2020).

### Peptide Sequencing of MS/MS Data

For *de novo* peptide sequencing with SMSNet (22), MS/MS spectra and precursor masses were extracted from raw MS files using ProteoWizard (32) with the following parameters: Peak Picking = Vendor for MS1 and MS2, Zero Samples = Remove for MS2, MS Level = 2-2, and the default Title Maker. Charge state deconvolution was not performed. The SMSNet-M model which treats carbamidomethylation of cysteine as fixed modification and oxidation of methionine as variable modification was used. Target amino acid-level false discovery rate was set at 5%. Precursor mass tolerance of 30 ppm was applied to discard identified peptides with high mass deviations. Partially identified peptides were searched against a UniProt (33) reference human proteome (downloaded August 2020) and a GRCh38 RefSeq (34) non-coding transcriptome (downloaded August 2020) to fill in the missing amino acids. From the transcriptome data, possible open reading frames that translate to at least 5 amino acids in length were considered.

For *de novo* sequencing-assisted database search with PEAKS version 8.5 (26), raw MS files were searched against a UniProt reference human proteome and reversed decoys. Cleavage enzyme specificity was set to none. Carbamidomethylation of cysteine, oxidation of methionine, and phosphorylation of serine, threonine, and tyrosine were set as variable modifications. A maximum of three modifications per peptide were allowed. Mass tolerances were set at 10 ppm for precursor mass and at 0.02 Da for fragment mass. Target peptide-level false discovery rate was set at 1%.

### HLA Binding Affinity and Binding Motif Analyses

For peptides identified from mono-allelic HLA peptidome experiments (8, 17), the binding affinities and the 9-mer binding motifs for the corresponding HLA alleles were predicted using NetMHCpan-4.1 (25) with default setting. For peptides identified from multi-allelic B-lymphoblastoid cell line, the binding affinities were predicted against all HLA class I or class II alleles present using NNAlign_MA (28). Predicted 9-mer binding motifs for each HLA class I allele were then clustered using GibbClusters (24). For each allele, the clustering was performed with number of clusters ranging from 1 to 5, with or without outlier detection, and with inter-cluster penalty parameter Λ ranging from 0.1 to 0.8. The optimal number of clusters was determined from the parameter setting with the highest Kullback-Liebler distance (KLD) as recommended by the authors (24). Information contents and the amino acid profiles of 9-mer binding motif clusters were visualized using Logomaker (35).

### HLA Binding Assay

The binding activities of selected 59 newly identified candidate antigens for HLA-B*14:02 (**Supplementary Table 3**) were assessed using the REVEAL MHC-peptide binding assay provided by ProImmune, Ltd. (Oxford, UK). Peptides were synthesized and quality checked using MALDI-TOF mass spectrometry by ProImmune, Ltd. (Oxford, UK). Binding activities were reported as percentage relative to the affinity of a positive control (a known high-affinity T cell epitope for HLA-B*14:02). According to the experiment report provided by the company, the standard error of the reported affinities is 3 percentage points.

## Data Availability Statement

Raw mass spectrometry data for the multi-allelic B-lymphoblastoid (BLCL1408-1038) peptidome are available at PXD028088. Visualizations of all identified motifs are available on FigShare at 10.6084/m9.figshare.16025226. Identified peptides from the multi-allelic B-lymphoblastoid (BLCL1408-1038) peptidome are available on FigShare at 10.6084/m9.figshare.19207386.

## Author Contributions

CS, TB, and PS analyzed HLA peptidomics data. PM performed experiments. SS, PS, and CS wrote the manuscript draft. SS and TP conceived and supervised the research. All authors contributed to and approved of the final manuscript.

## Funding

This work was supported by the Thailand Research Fund MRG6280189 (SS), the Grant for Special Task Force for Activating Research, Ratchadapisek Sompoch Endowment Fund, Chulalongkorn University (SS), the Grant for the Development of New Faculty Staff, Ratchadapisek Sompoch Endowment Fund, Chulalongkorn University (SS), the Thailand Research Fund for Career Development Grant RSA6280026 (TP), and Program Management Unit for Competitiveness Grant C10F630106 (TP). We would like to thank Prof. Vorasuk Shotelersuk, Department of Pediatrics, Faculty of Medicine, Chulalongkorn University, for mentorship under the Thailand Research Fund program and Dr. Pokrath Hansasuta, Department of Microbiology, Faculty of Medicine, Chulalongkorn University for insightful advice on HLA research.

## Conflict of Interest

The authors declare that the research was conducted in the absence of any commercial or financial relationships that could be construed as a potential conflict of interest.

## Publisher’s Note

All claims expressed in this article are solely those of the authors and do not necessarily represent those of their affiliated organizations, or those of the publisher, the editors and the reviewers. Any product that may be evaluated in this article, or claim that may be made by its manufacturer, is not guaranteed or endorsed by the publisher.
