# Supplementary material for: Unsupervised Mining of HLA-I Peptidomes Reveals New Binding Motifs and Potential False Positives in the Community Database
Source: Front Immunol. 2022 Mar 21;13:847756. doi: 10.3389/fimmu.2022.847756 (PMC8977642; doi:10.3389/fimmu.2022.847756)
Supplement: Supplementary file 1 [file DataSheet_1.docx]

Supplementary Tables

Supplementary Table 1 – List of all identified peptides together with predicted binding affinities and outlier detection results from mono-allelic peptidomics data of 88 HLA class I alleles

Supplementary Table 2 – Percentages of outlier peptides for HLA class I alleles with low percentage of tryptic peptides

Supplementary Table 3 – HLA-B*14:02 binding assay results for selected 59 peptides

Supplementary Table 4 – Number of peptides identified in replicate samples of multi-allelic B-lymphoblastoid cell line

Supplementary Table 5 – SMSNet and PEAKS identification results for multi-allelic B-lymphoblastoid cell line

Supplementary Figures


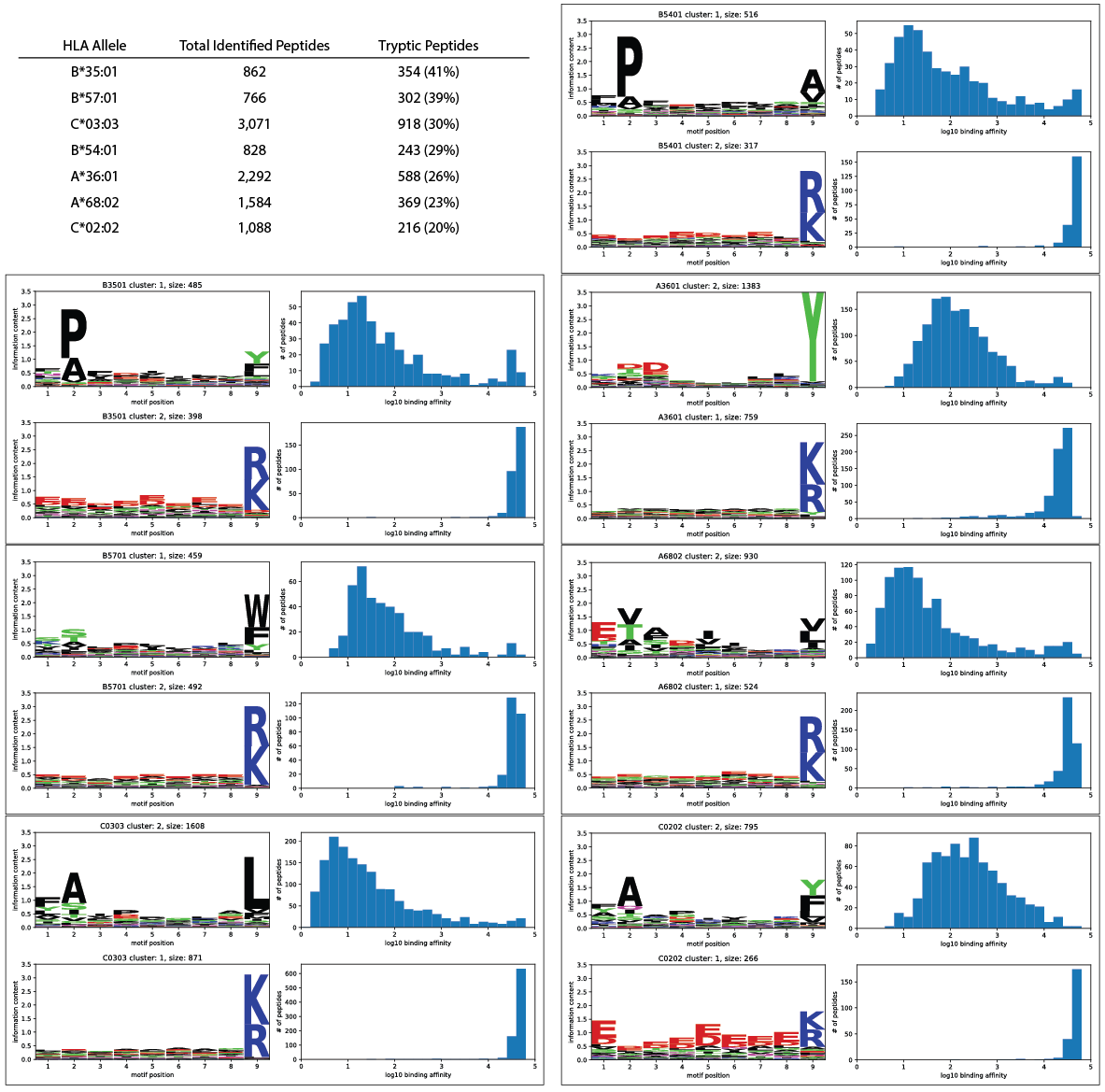


**Supplementary Figure 1** **– Extents of tryptic peptide contaminations in mono-allelic HLA peptidomics data**. Data for the top 7 alleles with more than 20% contaminations are shown. The table lists the numbers of all identified peptides and tryptic peptides for each allele. Each boxed region contains the 9-mer motif profiles and distributions of predicted binding affinity for each allele, sorted in the same order as shown in the table from left to right.


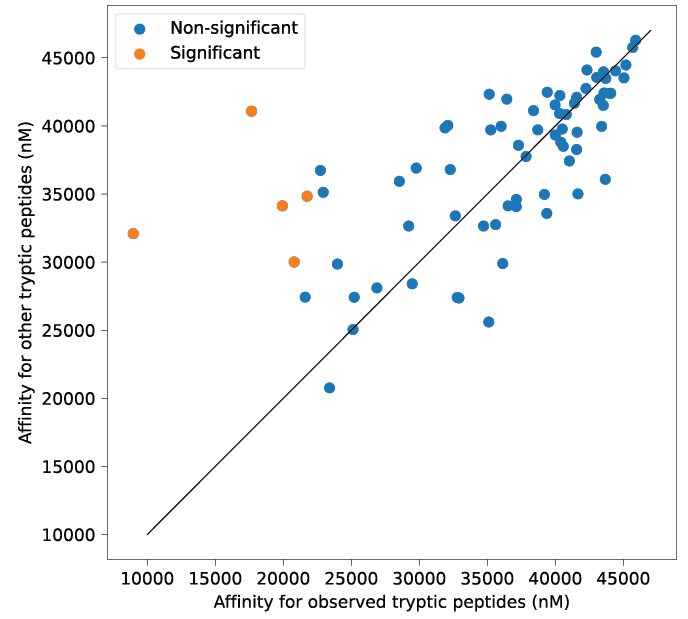


**Supplementary Figure 2 – HLA alleles do not exhibit stronger affinities toward observed tryptic peptides than toward random tryptic peptides**. Scatter plot shows the median predicted binding affinity (IC50, nM) between observed tryptic peptide-HLA allele pairs (x-axis) and that between random tryptic peptide-HLA pairs. Each data point represents one HLA allele. Higher IC50 value indicates lower affinity. Random tryptic peptides were selected from observed tryptic peptides in peptidomics data of all HLA alleles. Orange data points indicate the few HLA alleles that exhibit significantly stronger affinities toward tryptic peptides identified from the corresponding peptidomics data (Benjamini-Hochberg adjusted Mann-Whitney U test p-value < 0.05).


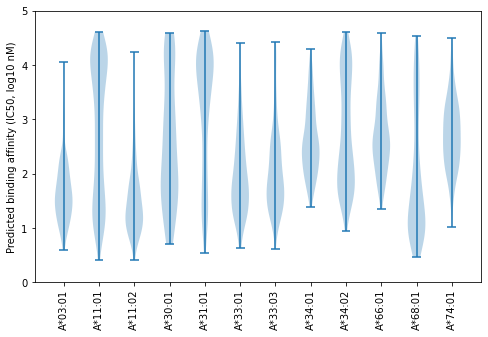
 **
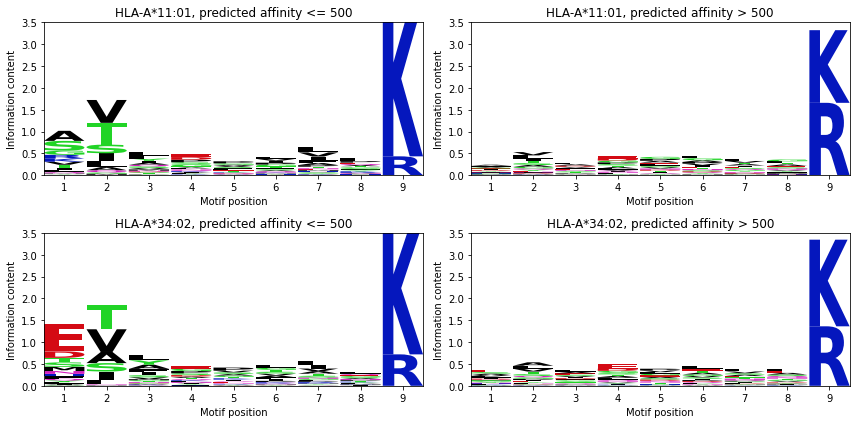
**


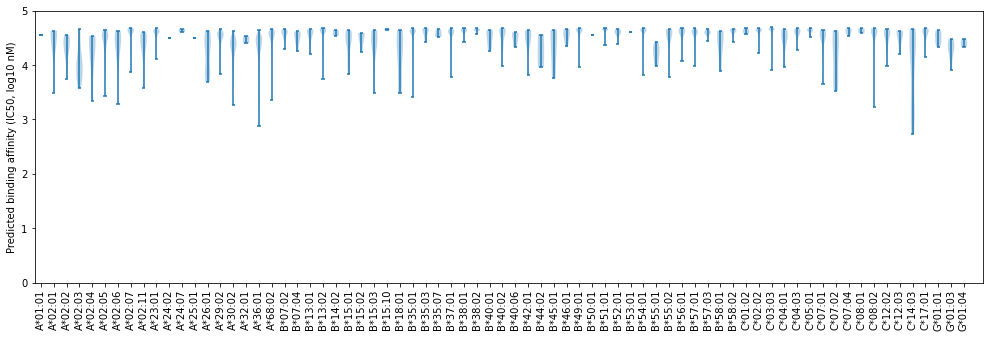
**Supplementary Figure 3** **– Predicted binding affinities from NetMHCpan can distinguish HLA alleles that can present tryptic peptides from other alleles**. Data from mono-allelic HLA peptidomics data were used. Violin plots showing the distribution of predicted binding affinities (IC50, log10 nM) for tryptic peptides identified in the peptidomics data. **Top left panel** shows the data for 12 alleles whose binding motifs do not end with an arginine or a lysine (HLA-A*03:01, HLA-A*11:01, HLA-A*11:02, HLA-A*30:01, HLA-A*31:01, HLA-A*33:01, HLA-A*33:03, HLA-A*34:01, HLA-A*34:02, HLA-A*66:01, HLA-A*68:01, and HLA-A*74:01). **Top right panel** shows the motif patterns and predicted binding affinities for HLA-A*11:01 and HLA-A*34:02 which exhibit bimodal distributions. The data for tryptic peptides with predicted IC50 ≤ 500 nM or >500 nM are shown separately. Predicted strong binders exhibit additional enrichments of specific amino acids at the first and second motif positions and higher preference for lysine at the ninth motif position while predicted weak binders do not. **Bottom panel** shows the predicted binding affinities of tryptics peptides from the other 76 alleles.


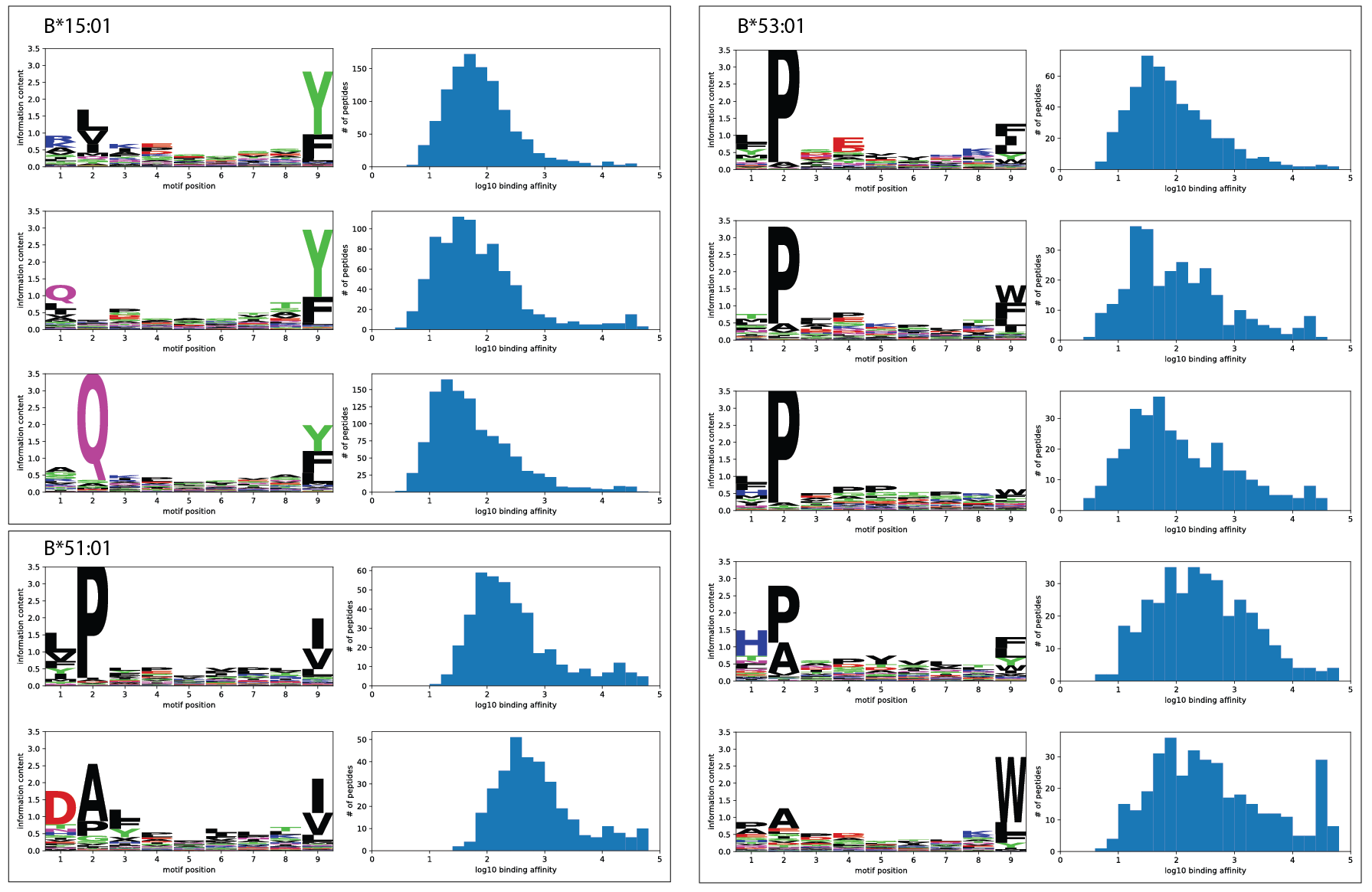


**Supplementary Figure 4** **– HLA alleles with multiple, clearly distinct motif specificities**. Data from mono-allelic HLA peptidomics data were used. Each boxed region contains motifs of the indicated HLA allele. Each 9-mer motif is shown alongside the distribution of predicted binding affinity (IC50, nM).


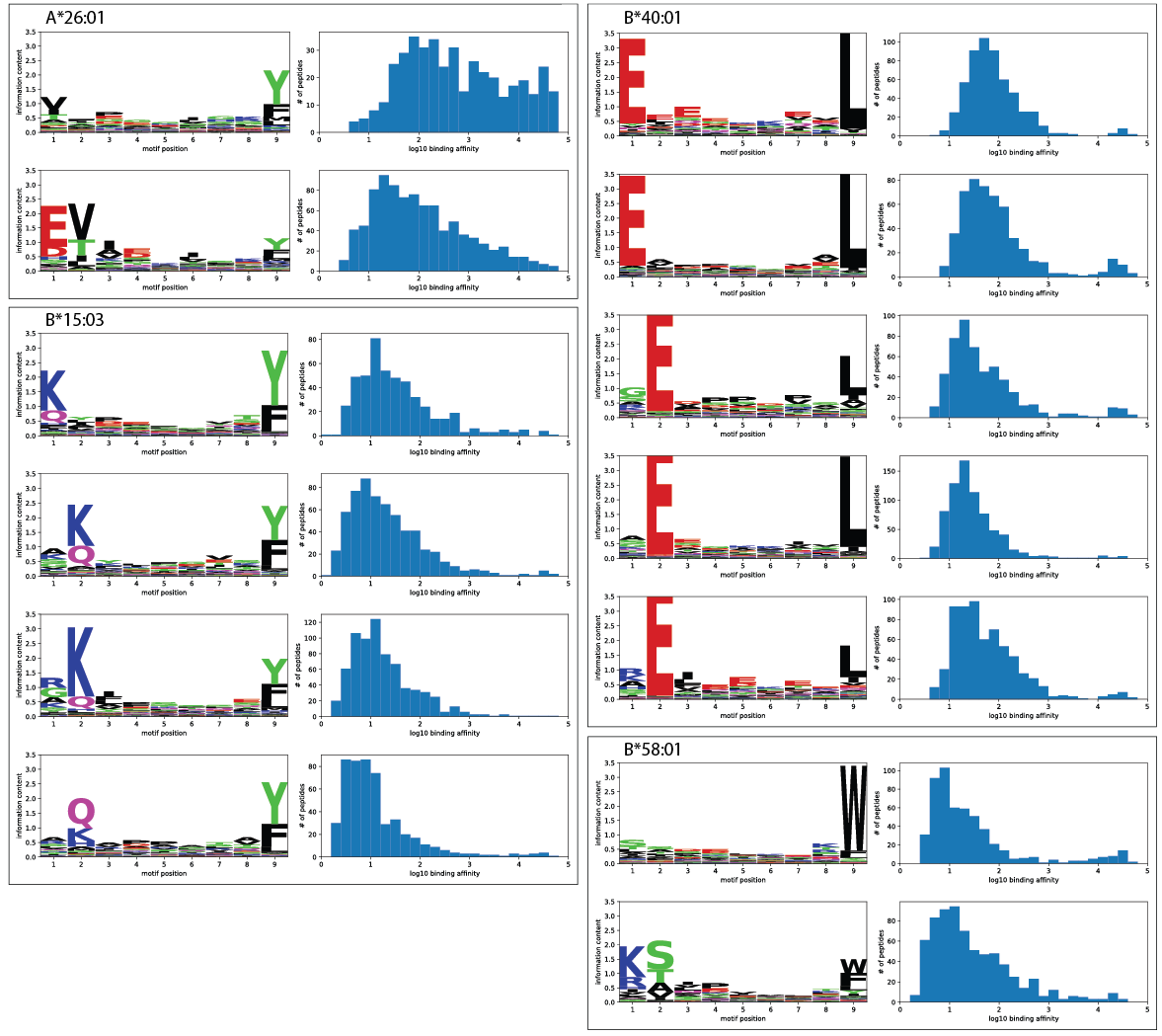


**Supplementary Figure 5** **– HLA alleles with multiple related motif specificities**. Data from mono-allelic HLA peptidomics data were used. Each boxed region contains motifs of the indicated HLA allele. Each 9-mer motif is shown alongside the distribution of predicted binding affinity (IC50, nM). These motifs possess similar anchor residues at the 2^nd^ position or shifted to the 1^st^ position. The 9-mer and 10-mer motif explanations for these patterns are illustrated in Supplementary Figure 6.


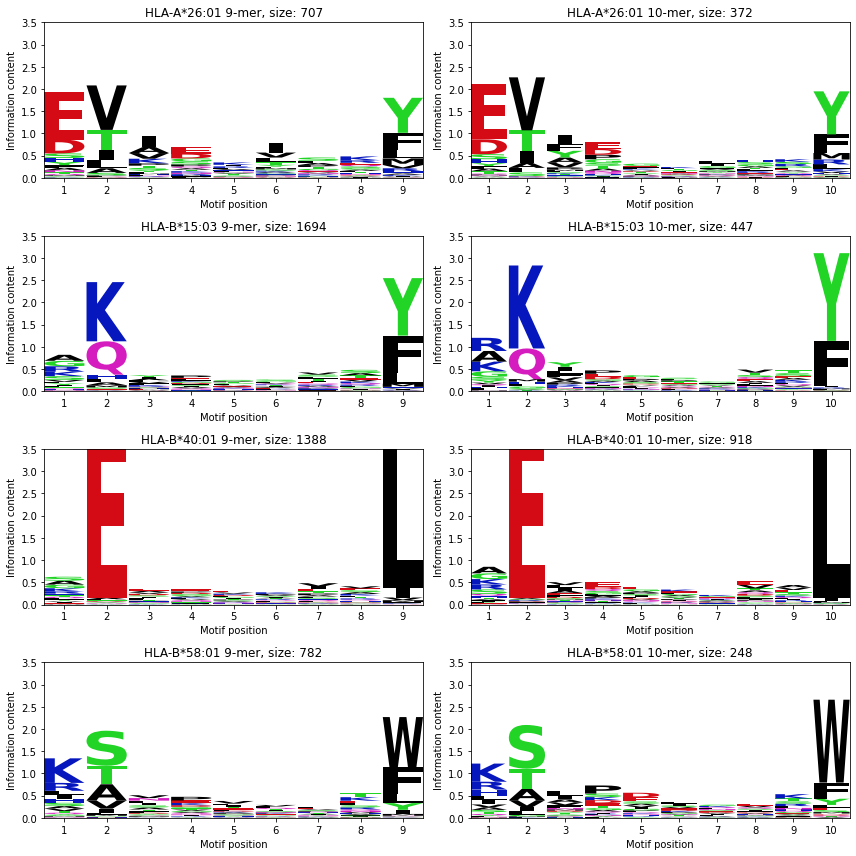


**Supplementary Figure 6** **– 9-mer and 10-mer motifs for HLA alleles with multiple related motif specificities**. Data from mono-allelic HLA peptidomics data were used. Each row shows 9-mer (left panel) and 10-mer (right panel) motifs of the indicated HLA allele.


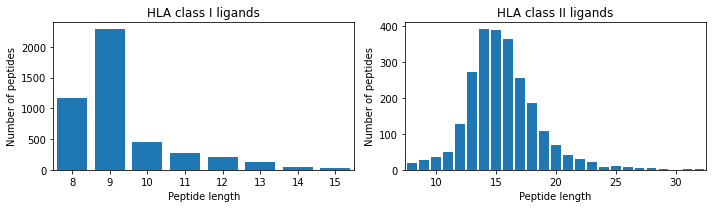


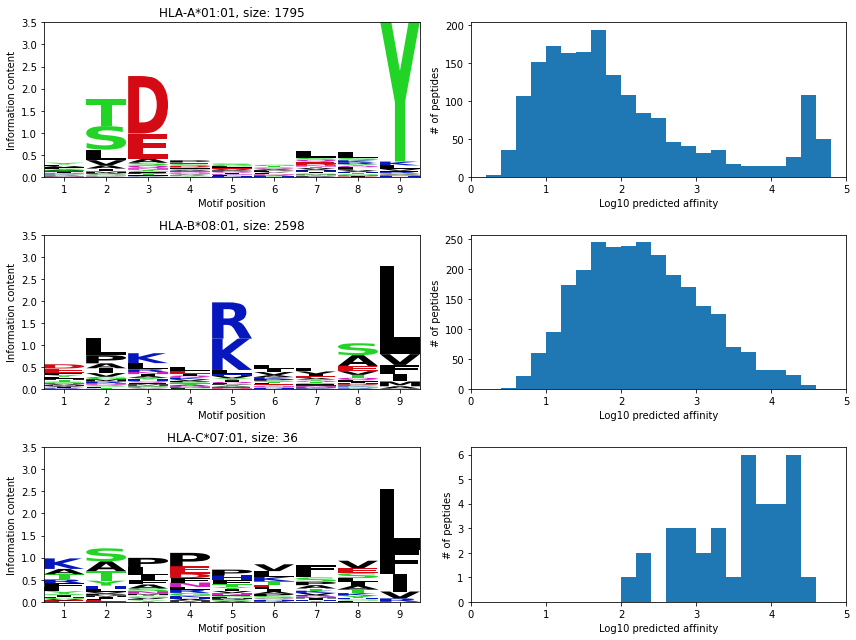


**Supplementary Figure 7** **– Characteristics of identified peptides and binding motifs from HLA peptidomics data of a multi-allelic B-lymphoblastoid cell line**. **The first row** shows the histograms of peptide lengths for peptides eluted from immunoaffinity purification with pan-HLA class I or pan-HLA class II antibody, respectively. **Bottom rows** show 9-mer motif alongside the corresponding distribution of predicted binding affinity (IC50, nM unit) for each HLA class I allele that is expressed in this cell line (HLA-A*01:01, HLA-B*08:01, and HLA-C*07:01). All identified peptides were included.


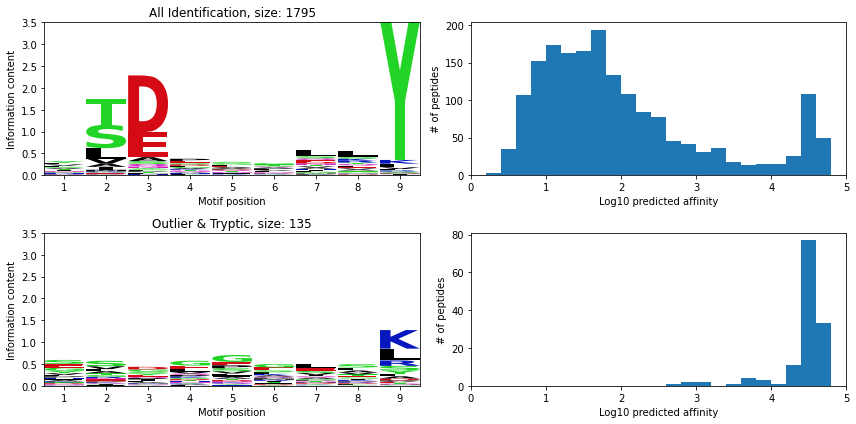


**Supplementary Figure 8** **– Motif clustering and predicted binding affinities for potential HLA-A*01:01 ligands identified from a multi-allelic B-lymphoblastoid cell line**. **The first row** shows the 9-mer motif profiles and histogram of the predicted binding affinity (IC50, nM unit) for all 1,795 peptides identified by SMSNet and PEAKS-DB. **The second row** shows the 9-mer motif profiles and histogram of the predicted binding affinity (IC50, nM unit) for 135 peptides flagged as either outlier or tryptic.


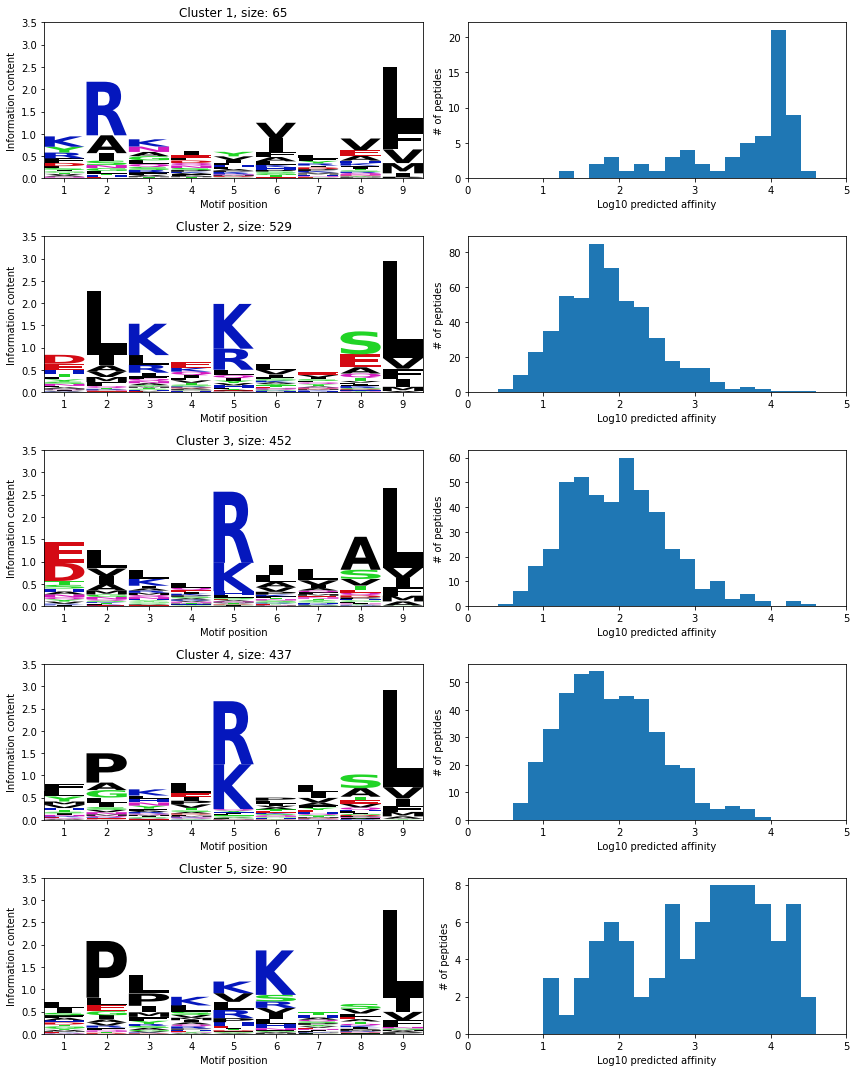


**Supplementary Figure 9** **– Motif clustering and predicted binding affinities for potential HLA-B*08:01 ligands identified from a multi-allelic B-lymphoblastoid cell line**. Each row shows the 9-mer motif profiles and histogram of the predicted binding affinity (IC50, nM unit) for each of the 5 clusters identified by GibbsCluster.


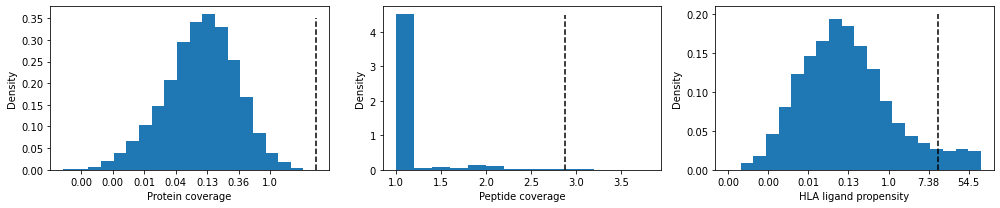

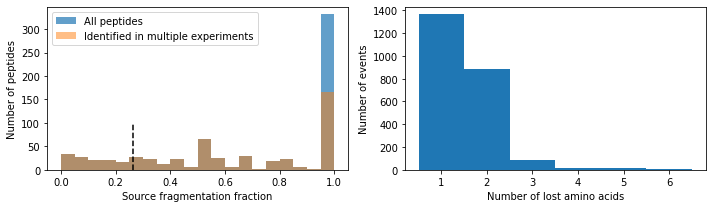

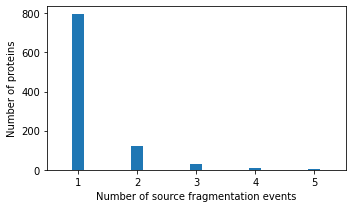


**Supplementary Figure 10** **– Application of best practices^1^ to identify potential proteolytic cleavages and in-source fragmentation products in mono-allelic HLA peptidomics data**. **Top row** shows the protein coverage, peptide coverage, and HLA ligand propensity (see reference^1^ for details on how these scores were calculated). Black vertical dashed lines indicate the 1% FDR cutoffs proposed in the original study. **Bottom row** shows the analysis of potential in-source fragmentation events. **The leftmost panel** shows the distribution of the Source Fragmentation Fraction score, which was defined as the fraction of times that a peptide could be explained as an in-source fragmentation of another co-eluding peptide in the same LC-MS/MS run (see reference^1^ for full description). Black vertical dashed lines indicate the 1% FDR cutoffs proposed in the original study. **The middle panel** shows the distribution of amino acids that were lost due to in-source fragmentation. **The rightmost panel** shows the distribution of the number of in-source fragmentation events per protein.

1. Fritsche J, Kowalewski DJ, Backert L, et al. Pitfalls in HLA Ligandomics-How to Catch a Li(e)gand. *Mol Cell Proteomics*. Jun 12 2021;20:100110. doi:10.1016/j.mcpro.2021.100110
